# High reproductive synchrony of *Acropora* (Anthozoa: Scleractinia) in the Gulf of Aqaba, Red Sea

**DOI:** 10.12688/f1000research.6004.1

**Published:** 2015-01-05

**Authors:** Jessica Bouwmeester, Michael L. Berumen

**Affiliations:** 1 Red Sea Research Center, King Abdullah University of Science and Technology, Thuwal, 23955-6900, Saudi Arabia

## Abstract

Coral spawning in the northern Gulf of Aqaba has been reported to be asynchronous, making it almost unique when compared to other regions in the world. Here, we document the reproductive condition of *Acropora* corals in early June 2014 in Dahab, in the Gulf of Aqaba, 125 km south of previous studies conducted in Eilat, Israel. Seventy-eight percent of *Acropora* colonies from 14 species had mature eggs, indicating that most colonies will spawn on or around the June full moon, with a very high probability of multi-species synchronous spawning. Given the proximity to Eilat, we predict that a comparable sampling protocol would detect similar levels of reproductive synchrony throughout the Gulf of Aqaba consistent with the hypothesis that high levels of spawning synchrony are a feature of all speciose coral assemblages.

## Observation

Multi-species synchronous spawning of scleractinian corals is a feature reported from almost all speciose coral assemblages ( Baird *et al.*, 2009a; Baird *et al.*, 2010; Raj & Edwards, 2010), including the Arabian Sea ( Baird *et al.*, 2014), and the Red Sea ( Bouwmeester *et al.*, 2011; Bouwmeester *et al.*, 2014; Hanafy *et al.*, 2010). A notable exception is at Eilat, on the Israeli coast, in the Gulf of Aqaba, where spawning is described as asynchronous with different species spawning in different seasons, on different months, and at different stages of the lunar cycle, with no overlap in spawning between species ( Shlesinger & Loya, 1985; Shlesinger *et al.*, 1998).

**Figure 1.**
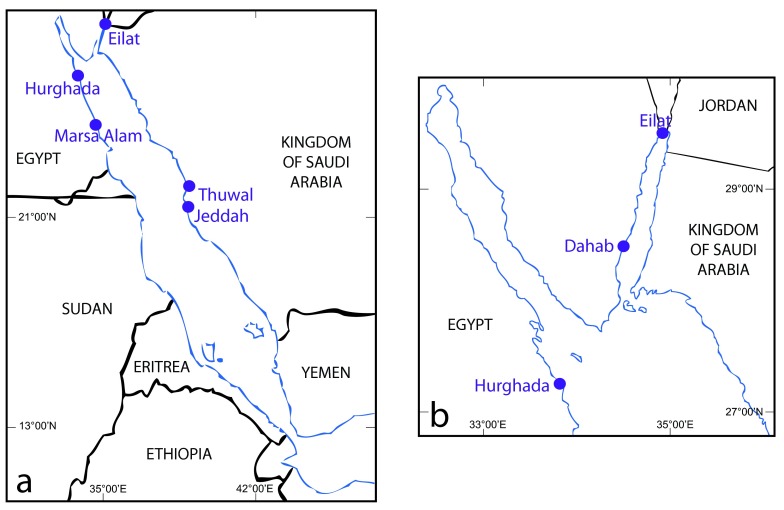
Map of **a** the Red Sea, showing the location of previous work on scleractinian coral spawning in the Red Sea, and **b** the Gulf of Aqaba, showing Dahab, our study site.

Here, we quantify the reproductive synchrony of *Acropora* corals in Dahab, on the Egyptian coast of the Gulf of Aqaba, 125 km south of Eilat, Israel ( Figure 1). Among Red Sea reef habitats, fringing reefs in the Gulf of Aqaba support distinct coral assemblages with high cover and diversity of hard corals ( DeVantier *et al.*, 2000).

**Figure 2.**
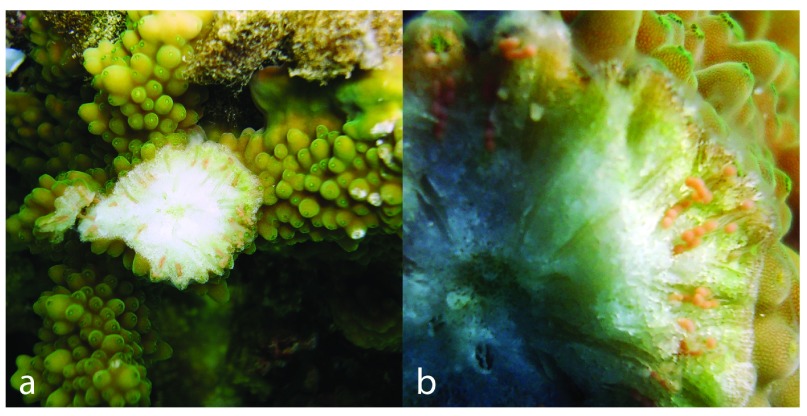
**a** Exposed oocytes in a mature colony of *Acropora variolosa* **b** close-up of pigmented oocytes.

The reproductive condition of 90 colonies from 15 *Acropora* species was assessed at two dive sites in Dahab, Um Sid (28° 25’14.16”N, 34° 27’27.52”E) and Eel Garden (28° 30’19.21”N, 34° 31’15.58”E), from the 2 ^nd^ to the 4 ^th^ of June 2014, a week before the full moon that month ( Table 1). *Acropora* colonies at 1–10m depth were examined on snorkel by breaking 1–3 coral branches below the sterile apical zone to expose the developing oocytes. Colonies were recorded as mature when oocytes were visible and pigmented ( Figure 2), immature when oocytes were visible and white, and empty when oocytes were too small to see with the naked eye or absent (following Baird *et al.*, 2002). Colonies with mature oocytes are highly likely to spawn close to the night of the next full moon (in this case, the June full moon), whereas colonies with immature eggs are likely to spawn on or around a full moon one or two months later (in this case the July or August full moon). Colonies without oocytes have either already spawned or are unlikely to do so for at least three months.

**Table 1.** Percentage (%) of *Acropora* colonies with mature, immature, and no visible oocytes, on the 2–4 June 2014, in Dahab, Egypt, in the Gulf of Aqaba, Red Sea. n: number of sampled colonies.

Species	% mature	% immature	% empty	n
*Acropora aculeus*	100	0	0	4
*Acropora cytherea*	22	11	67	9
*Acropora digitifera*	100	0	0	6
*Acropora eurystoma*	86	0	14	7
*Acropora gemmifera*	100	0	0	7
*Acropora horrida*	0	0	100	1
*Acropora humilis*	50	25	25	8
*Acropora lutkeni*	75	0	25	4
*Acropora microclados*	100	0	0	4
*Acropora monticulosa*	100	0	0	7
*Acropora nasuta*	86	0	14	7
*Acropora polystoma*	80	0	20	10
*Acropora* cf *samoensis*	100	0	0	6
*Acropora valida*	100	0	0	3
*Acropora variolosa*	57	0	43	7
**Total**	**78**	**3**	**19**	**90**

Seventy-one percent of *Acropora* colonies had mature oocytes and an additional three percent had immature oocytes ( Table 1). No oocytes were observed in the remaining colonies. Fourteen out of 15 species had at least one colony with mature eggs, and in seven of those species, 100% of the sampled colonies had mature eggs ( Table 1).

The reproductive condition in the *Acropora* assemblage at Dahab in June is very similar to that estimated in *Acropora* assemblages on the Egyptian coast of the northern Red Sea, where 85% of colonies from 12 species had mature oocytes in Marsa Alam in April 2008 and 99% of colonies from 17 species had mature oocytes in Hurghada in April 2009 ( Hanafy *et al.*, 2010). Subsequent sampling in both years revealed the absence of oocytes in all but one of these species, indicating that spawning had occurred sometime in the previous couple of weeks, most likely around the full moon of April ( Hanafy *et al.*, 2010). Nighttime observations in 2012 in Hurghada revealed spawning of 12 *Acropora* species over two consecutive nights around the full moon of May ( Kotb, 2012). Similarly, 13 *Acropora* species in Thuwal, central Red Sea ( Figure 1a), were observed to spawn together on the same night, both in April 2011 and in April 2012, following initial reproductive surveys which revealed 65% of mature *Acropora* colonies from 9 species in 2011 and 39% of mature *Acropora* colonies from 16 species in 2012 ( Bouwmeester *et al.*, 2014). The high percentage of species and colonies with mature oocytes in Dahab one week before the June full moon strongly suggests they will spawn synchronously as observed in Thuwal in the central Red Sea ( Bouwmeester *et al.*, 2011; Bouwmeester *et al.*, 2014) and in Hurghada in the northern Red Sea ( Hanafy *et al.*, 2010; Kotb, 2012). Broadcast spawning of corals in most locations of the Indo-Pacific occurs as sea surface temperatures are increasing or when temperatures are close to their annual maxima ( Baird *et al.*, 2009a). In Dahab, waters start warming in the months of March-April, rising from 21–22°C to a maximum of 26–27°C in the month of August ( Cornils *et al.*, 2007; Plähn *et al.*, 2002). Spawning in June most likely occurs when temperatures are ~24–25°C, possibly an optimum temperature for spawning and early larval development in the Gulf of Aqaba.

The month of spawning of *Acropora* species in the Gulf of Aqaba is two months later than in the northern and central Red Sea, where most *Acropora* spawn in April ( Bouwmeester *et al.*, 2014; Hanafy *et al.*, 2010). This one or two-month offset is not surprising due to the difference in local temperature regimes and is similar to the latitudinal pattern observed along the east coast of Australia and from the Philippines to Japan ( Baird *et al.*, 2009b). Spawning in Dahab does not seem to occur before the waters reach 24–25°C, suggesting that a minimal temperature threshold is required during the warming of surface waters for spawning. In the central Red Sea, those temperatures are reached in March-April, and indeed multi-species spawning of *Acropora* has been recorded in April at 25–27°C ( Bouwmeester *et al.*, 2014).

Our data from Dahab match the data from Eilat ( Shlesinger & Loya, 1985) for the timing of *Acropora* spawning in the Gulf of Aqaba, however, the larger number of *Acropora* species examined in the present study allows us to understand reproductive synchrony within this genus much more effectively. Indeed, we predict that with a comparable sampling protocol, similar levels of *Acropora* reproductive synchrony would be detected at Eilat, only 125 km north of Dahab, and would support the hypothesis that high levels of spawning synchrony are a feature of all speciose coral assemblages ( Guest *et al.*, 2005). The length of the scleractinian reproductive season can be established by sampling distantly related species from the coral assemblage, which in Eilat lasts four months for broadcast spawning species ( Guest *et al.*, 2005; Shlesinger & Loya 1985; Shlesinger *et al.*, 1998), but sampling closely related species such as *Acropora* species will determine whether overlap in spawning occurs and will allow estimation of the level of synchrony in the assemblage.

## References

[ref-1] Baird AH Marshall PA Wolstenholme JK: Latitudinal variation in the reproduction of *Acropora* in the Coral Sea. *Proc 9th Int Coral Reef Symp.* 2002;1:385–389. Reference Source

[ref-2] Baird AH Guest JR Willis BL: Systematic and biogeographical patterns in the reproductive biology of scleractinian corals. *Annu Rev of Ecol Evol Syst.* 2009a;40:551–571. 10.1146/annurev.ecolsys.110308.120220

[ref-3] Baird AH Birrell CL Hughes TP: Latitudinal variation in reproductive synchrony in *Acropora* assemblages: Japan vs. Australia. *Galaxea.* 2009b;11(2):101–108. 10.3755/galaxea.11.101

[ref-4] Baird AH Kospartov MC Purcell S: Reproductive synchrony in *Acropora* assemblages on reefs of New Caledonia. *Pac Sci.* 2010;64(3):405–412. 10.2984/64.3.405

[ref-5] Baird AH Abrego D Howells EJ: The reproductive season of *Acropora* in Socotra, Yemen. [v2; ref status: indexed, http://f1000r.es/392] *F1000Res.* 2014;3:78. 10.12688/f1000research.3846.2 25075295 PMC4095572

[ref-6] Bouwmeester J Khalil MT De La Torre P: Synchronous spawning of *Acropora* in the Red Sea. *Coral Reefs.* 2011;30(4):1011–1011. 10.1007/s00338-011-0796-5

[ref-7] Bouwmeester J Baird AH Chen CJ: Multi-species spawning synchrony within scleractinian coral assemblages in the Red Sea. *Coral Reefs.* 2014;1–13. 10.1007/s00338-014-1214-6

[ref-8] Cornils A Schnack-Schiel S Al-Najjar T: The seasonal cycle of the epipelagic mesozooplankton in the northern Gulf of Aqaba (Red Sea). *J Mar Syst.* 2007;68(1–2):278–292. 10.1016/j.jmarsys.2007.01.001

[ref-9] DeVantier L Turak E Al-Shaikh K: Coral communities of the central-northern Saudi Arabian Red Sea. *Fauna of Arabia.* 2000;18:23–66. Reference Source

[ref-10] Guest JR Baird AH Goh BPL: Seasonal reproduction in equatorial reef corals. *Inverteb Reprod Dev.* 2005;48:207–218. 10.1080/07924259.2005.9652186

[ref-11] Hanafy MH Aamer MA Habib M: Synchronous reproduction of corals in the Red Sea. *Coral Reefs.* 2010;29(1):119–124. 10.1007/s00338-009-0552-2

[ref-12] Kotb MMA: Reef Check spotlight: Synchronized coral spawning in the Red Sea. *The Transect Line.* 2012;2 Reference Source

[ref-13] Plähn O Baschek B Badewien TH: Importance of the Gulf of Aqaba for the formation of bottom water in the Red Sea. *J Geophys Res.* 2002;107(C8):22-1–22-18. 10.1029/2000JC000342

[ref-14] Raj KD Patterson J: Observations on the reproduction of *Acropora* corals along the Tuticorin coast of the Gulf of Mannar, Southeastern India. *Indian J Mar Sci.* 2010;39(2):219–226. Reference Source

[ref-15] Shlesinger Y Loya Y: Coral community reproductive patterns: Red Sea versus the Great Barrier Reef. *Science.* 1985;228(4705):1333–1335. 10.1126/science.228.4705.1333 17799121

[ref-16] Shlesinger Y Goulet T Loya Y: Reproductive patterns of scleractinian corals in the northern Red Sea. *Mar Biol.* 1998;132(4):691–701. 10.1007/s002270050433

