# Supplementary material for: High reproductive synchrony of Acropora (Anthozoa: Scleractinia) in the Gulf of Aqaba, Red Sea
Source: F1000Res. 2015 Jan 5;4:2. [Version 1] doi: 10.12688/f1000research.6004.1 (PMC4304220; doi:10.12688/f1000research.6004.1)
Supplement: Peer review history

# Peer review: Referee response for version 1

**DOI:** 10.5256/f1000research.6425.r7208

**Published:** 2015-01-16

**Authors:** Andrew G. Bauman

**Affiliations:** 1 Department of Biological Sciences, Experimental Marine Ecology Laboratory, National University of Singapore, Singapore, Singapore

This article presents information on the reproductive synchrony of 15 *Acropora* species in the southern Gulf of Aqaba, Red Sea. This is a sound study that used well-established sampling techniques, and provides adequate details for the methods, results and findings. Their results showed a high proportion of *Acropora *colonies (78%) sampled prior to the full moon in June had mature oocytes and suggest that most colonies will spawn in June, with a high probability of multi-species spawning. Most interestingly, their data matched previously spawning records from Eilat, 125km north of their site, providing additional evidence that high levels of spawning synchrony are likely a feature of all speciose coral assemblages.

NOTE: While I agree with the authors that it is likely that the colonies with mature (pigmented) oocytes are generally considered to spawn on, or shortly after, the subsequent full moon, conducting follow up surveys in the subsequent months (i.e., July and August) would confirm whether colonies are in fact releasing all of their gametes. In a similar reproductive study conducted in the neighboring Persian Gulf ( Bauman et al. 2011) mature colonies were found over four consecutive months suggesting that some colonies are either not releasing mature gametes or that some colonies are spawning twice.

I have read this submission. I believe that I have an appropriate level of expertise to confirm that it is of an acceptable scientific standard.

# Peer review: Referee response for version 1

**DOI:** 10.5256/f1000research.6425.r7207

**Published:** 2015-01-07

**Authors:** Jean Kenyon

**Affiliations:** 1 Hawaiian and Pacific Islands National Wildlife Refuge Complex, U.S. Fish and Wildlife Service, Oahu, HI, USA

The research uses an accepted method of assessing reproductive condition in *Acropora *colonies. The article clearly and concisely reports the location, species, and number of colonies sampled and provides background context on other coral reproductive sampling in the Red Sea, which combine to bring the reader to the same conclusions as the authors regarding multi-species spawning synchrony in this region. One hopes the authors will find the resources to test their hypothesis that similar levels of *Acropora* reproductive synchrony would be detected at Eilat using a comparable sampling protocol. Overall, the writing (including title, abstract, and main body of text) is succinct and provides an appropriate level of detail.

I have read this submission. I believe that I have an appropriate level of expertise to confirm that it is of an acceptable scientific standard.
